# Unbalanced Oxidant-Antioxidant Status: A Potential Therapeutic Target for Coronary Chronic Total Occlusion in Very Old Patients

**DOI:** 10.1155/2016/4910829

**Published:** 2016-12-01

**Authors:** Xia Li, Youdong Hu, Fenglin Zhang, Ying Chen, Hualan Zhou, Dianxuan Guo, Qingna Zhao

**Affiliations:** Department of Geriatrics, The Affiliated Huai'an Hospital of Xuzhou Medical University, Huai'an, Jiangsu 223002, China

## Abstract

Unbalanced oxidant and antioxidant status played an important role in myocardial infarction. The present study was a clinical trial combined preclinically with targeted agent against cardiovascular injuries and ischemia in vivo model. We tried to confirm the association of unbalanced oxidant and antioxidant status with coronary chronic total occlusion (CTO) in 399 very old patients (80~89 years) and investigated the potential therapeutic value of purified polysaccharide from endothelium corneum gigeriae galli (PECGGp). We analyzed levels of circulating superoxide dismutase 3 (SOD3), nitric oxide (NO), endothelial nitric oxide synthase (eNOS), and malondialdehyde (MDA) in very old patients with coronary CTO. Levels of SOD3, NO, eNOS, and MDA in the cardiac tissue were measured in myocardial infarction rats. Levels of SOD3, eNOS, and NO were lowered (*p* < 0.001) and levels of MDA were increased (*p* < 0.001). PECGGp treatment increased levels of SOD3, eNOS, and NO (*p* < 0.01) in cardiac tissue, while decreasing levels of MDA (*p* < 0.01). PECGGp may suppress unbalanced oxidant and antioxidant status in infarcted myocardium by inhibiting levels of MDA and elevating NO, eNOS, and SOD3 levels. PECGGp could be considered as a potential therapeutic agent for coronary CTO in very old patients.

## 1. Introduction

The relationship between impairment of nitric oxide (NO) signaling pathway and myocardial infarction risk has been identified [1–3]. Decreased NO bioactivity and elevated reactive oxygen species levels contributed to impairment of coronary arteries [4]. Thus, NO regulation can be a novel therapeutic target for protecting against myocardial infarction and congestive heart failure [5, 6].

The genetic deficiency of NOS can cause heart failure [5]. Enhanced external counterpulsation treatment for coronary heart disease patients inhibited the development of atherosclerotic lesions by stimulating NOS and NO signaling pathways [7, 8]. NOS in the myocardium had displayed novel molecular targets by which NO regulated nitroso-redox balance. NOS could be a treatment option in patients with heart diseases [9, 10].

Intervention via suppression of reactive oxygen species generation or enhancement of endogenous antioxidant enzymes may limit the infarct size and attenuate myocardial dysfunction [11–13]. Elevating MDA levels in patients with coronary heart disease impaired NO production and MDA levels were remarkably elevated in congestive heart failure patients [14–17]. The studies suggested MDA concentrations were associated with thin-cap fibroatheroma, complex atherosclerotic plaque, and atherosclerotic plaque instability and they are the main cause of myocardial infarction. Anti-MDA could be useful for developing potential antiatherosclerosis vaccine [18].

Superoxide dismutase (SOD) can regulate reactive oxygen species levels and significantly increase in the NO bioactivity under oxidative stress. The expression of the antioxidant enzyme SOD reduced cardiovascular injury and played a vital role in antisuperoxide formation, antioxidative stress damage, and artery angiogenesis. Oxidative stress by elevating reactive oxygen species had been involved in atherosclerosis and heart failure by inhibiting bioactivity of NO in the vascular walls [19–22]. SOD was a major antioxidative enzyme in the walls of arteries and heavily damaged in coronary heart disease patients. The decreasing activity of SOD contributed to a reduction in NO bioavailability and led to high levels of oxidative stress in coronary heart disease patients. The decreased NO bioavailability may promote development of coronary artery atherosclerosis [23, 24]. Gene transfer of SOD promoted aortic endothelial repair and prevented atherogenesis. SOD had been considered as a main modulator of NO bioactivity and may have the potential therapeutic effects in preventing or reversing cardiovascular damage and ischemic heart failure. However, a novel natural SOD activator under oxidative stress is even more worthy [25, 26].

Patients with coronary heart disease who underwent primary percutaneous coronary intervention were more often of older age. The Occluded Artery Trial and The Synergy between Percutaneous Coronary Intervention With Taxus and Cardiac Surgery (SYNTAX) trial have demonstrated that primary percutaneous coronary intervention does not decrease the incidence of major adverse cardiac events and may lead to ischemic injury to the myocardium with increasing the rates of recurrent myocardial infarction and repeating coronary revascularizations in the patients with coronary chronic total occlusion (CTO). Coronary artery bypass graft surgery was more invasive than primary percutaneous coronary intervention and was executed in older patients with more severe coronary heart disease [27–30].

Our findings suggested that intracoronary infusion of human umbilical cord mesenchymal stem cells ameliorated left ventricular ejection fraction and decreased infarct size remarkably in very old patients with coronary CTO [31, 32]. However, primary percutaneous coronary intervention procedures took longer and had the risks of radiation skin injury and acute kidney injury. Primary percutaneous coronary intervention procedures of coronary CTO involved major risk factors for artery dissection and perforation of artery dissection and cardiac tamponade was a serious complication of primary percutaneous coronary intervention [28]. Therefore, further studies are needed to evaluate the potential novel noninvasive therapy for coronary CTO in very old patients. The present study aimed to demonstrate the association of unbalanced oxidant and antioxidant status with coronary CTO in very old patients and investigate potential therapeutic and preventive values of purified polysaccharide from endothelium corneum gigeriae galli (PECGGp).

## 2. Subjects and Methods 

### 2.1. Study Population

The study was approved by Xuzhou Medical University and University Affiliated Hospital Ethical Review Board according to the Chinese law and regulations, and informed consent was obtained from the participants according to the Declaration of Helsinki. We studied 399 consecutive very old patients who underwent coronary angiography (232 men; 84.7 ± 5.10 years) from 1 January 2003 to 31 December 2011. The inclusion criteria included (1) age ≥ 80 years, (2) at least single vessel disease or one coronary CTO, and (3) symptomatic angina and/or ischemic heart disease.

Exclusion criteria were (1) recent use of antioxidant supplement, (2) uncontrolled hypertension, (3) acute myocardial infarction, (4) cardiopulmonary resuscitation, (5) severe ventricular arrhythmias, (6) acute coronary syndrome, (7) cardiogenic shock, (8) atrial fibrillation, (9) acute heart failure, (10) stroke, (11) immune-mediated diseases, (12) acute and chronic liver diseases, (13) hematologic disorders, (14) malignant tumours, (15) severe renal impairment, (16) acute and chronic inflammatory diseases, (17) severe iodinated contrast material reactions, (18) hyperthyroidism, (19) severe obstructive lung disease, (20) dialysis, (21) serious anemia, and (22) severe peripheral arterial disease.

### 2.2. Study Protocol

The patients were categorized as having control (CON) group (*n* = 65), single vessel disease (SVD) group (*n* = 114), multivessel disease (MVD) without CTO group (*n* = 93), MVD with one CTO group (*n* = 71), and MVD with multiple CTO group (*n* = 56). Coronary artery lesion with a diameter stenosis of <50% was included in CON group. SVD was defined as just one coronary artery stenosis ≥70%. MVD was defined as > one major coronary artery, stenosis of ≥70% or left main stenosis of ≥50%. The diagnosis of CTO was based on a total occlusion in a non-infarct-related artery, collateralization of the distal vessel, 100% luminal diameter stenosis with thrombolysis in myocardial infarction flow grade 0, and duration of obstruction for ≥3 months. The stump of the CTO can be defined as tapered. The duration was estimated from the last showing CTO in very old patients who underwent coronary angiograms, myocardial infarction, or acute coronary syndrome, or exertional angina and the presence of coronary collateral vessels on angiography [30, 33]. The left ventricular ejection fraction data were collected from 2-dimensional echocardiogram. The left ventricular ejection fraction was classified as follows: grade I (≥55%); grade II (40% to 54%); grade III (30% to 39%); and grade IV (<30%) [34].

### 2.3. Coronary Artery Angiography and Echocardiographic Studies

The patients received 300 mg aspirin loading dose along with 600 mg clopidogrel after the collection of blood samples. Coronary angiography was done through the femoral and or radial arteries by antegrade approach. Angiography of bilateral arteries was performed in some patients. Coronary angiographic analyses were performed with the dedicated coronary bifurcation computer system (Qangio® XA, 7.3, MEDIS, Medical Imaging System BV, Leiden, Netherlands). The bifurcation lesions were divided into 4 fragments: the central bifurcation segment, the distal segment of main vessel, the proximal segment main vessel, and the side branch [35–37]. Coronary artery angiograms were analyzed quantitatively by 2 independent cardiologists with experience and blind to the identities and clinical characteristics of all subjects. Discrepancies were resolved by consensus of a third cardiologist. According to the American Society of Echocardiography Standards, a complete Doppler echocardiography study was carried out with electrocardiograms synchronized by two independent cardiologists who were blinded to the clinical trial data of all subjects as described [31].

### 2.4. Measures of Plasma NO and eNOS

The patients' blood was drawn into the heparinized test tubes, centrifuged (1000 ×g for 10 min at 4°C), and stored at −70°C. The nitrates were reduced to nitrites by nitrate reductase and the plasma levels of NO were quantified using UV spectrophotometry (545 nm) followed by a PicoGreen measurement. The results were expressed as *μ*mol/L [38].

eNOS levels were expressed as pg/mL and the range of this assay was 0.156 to 1000 pg/mL. EDTA-anticoagulated blood samples were obtained at fasting. The blood samples were centrifuged at 1200 rpm for 10 minutes at 4°C and stored at −80°C for assays. Each blood sample was analyzed duplicately, and the overall intra-assay coefficient of variation was best calculated to be 3.6%. All blood samples were measured in the central laboratory and the plasma concentrations of eNOS were assayed by Sigma's Sandwich ELISA kit according to the manufactures' instructions [39].

### 2.5. Determination of MDA and SOD3 Levels

Plasma MDA concentrations were determined by a high performance liquid chromatography system with fluorometric detection (Shimadzu, Japan) and excitation was performed at 532 nm. MDA in the samples was reacted with a solution of 2-thiobarbituric acid by incubating for two hours at 60°C. Plasma MDA levels were expressed as nmol/L [40]. The blood samples for SOD3 assays were centrifuged at 3000 rpm for 15 minutes. The plasma SOD3 activity was determined by using enzyme-linked immunosorbent assay according to the manufacturer's instructions. The assay kit for SOD3 was provided by RANSOD-Randox, UK. The results from the assay were expressed as U/mL [41].

### 2.6. Preparation and Purification of Polysaccharide from Endothelium Corneum Gigeriae Galli

Endothelium corneum gigeriae galli was prepared and purified according to our published procedures [42]. Briefly, endothelium corneum gigeriae galli was grinded into powders and the powders were defatted to form pretreated powders. The pretreated powders of 10 g were drawn out using distilled water and the supernatants were deproteinated and centrifugated to afford water solution endothelium corneum gigeriae galli. The endothelium corneum gigeriae galli was purified with diethylaminoethyl cellulose 52 chromatography and Sephadex® G-100. The water solution of endothelium corneum gigeriae galli was added to column of diethylaminoethyl cellulose 52. The carbohydrates eluting from the column were determined using the phenol-sulfuric acid method. One water polysaccharide fraction was collected and further purified to obtain purified polysaccharide from endothelium corneum gigeriae galli (PECGGp**)**.

### 2.7. Evaluation of Cardioprotective Activities of PECGGp

According to our published procedures [42], adult male Sprague-Dawley rats were randomized into 5 groups (each *n* = 10): Group A (control group), Group B (PECGGp-untreated model group), Group C (positive control group), Group D (PECGGp low-dose group), and Group E (PECGGp high-dose group). Group A was administrated gastric gavage of 0.9% NaCl (25 mL/per kg body weight) every day. Group B was given gastric gavage of 0.9% NaCl (25 mL/per kg body weight) every day. Group C was treated by gastric gavage with propranolol 30 mg/kg/day. Group D and E were given gastric gavage with daily doses of PECGGp (80 and 240 mg/kg), respectively. After 14 days, all rats were treated subcutaneously with isoproterenol (5 mg/kg/day) for two consecutive days to develop myocardial infarction. Left ventricular ejection fraction was assessed using nuclear magnetic resonance imaging.

### 2.8. Immunohistochemistry Assessments of SOD3, NO, eNOS, MDA, Syndecan-1, and Nuclear Factor Erythroid-2-Related Factor 2 (Nrf2) in Postinfarct Rat Myocardium

The homogenate of rat myocardium was dissociated in ice cold homogenization buffer (Phosphate buffer, 0.05 M pH 7.4) by laboratory homogenate machine. NO and MDA levels were determined by the reduction of nitrate and colorimetric methods using thiobarbituric acid. eNOS and SOD3 activities were assessed with xanthine oxidase and chemical chromatometry methods according to the manufacturer's instructions [42]. Cardiac tissue cyclic GMP levels were measured by an enzyme immunoassay test-system (R&D Systems, Inc. Minneapolis, MN). Myocardium samples were reacted with a rabbit Nrf2 polyclonal antibody (1 : 100; Santa Cruz Biotechnology, Inc). Immunohistochemistry for syndecan-1 was performed on myocardium sections and the sections were stained with antibodies specific to syndecan-1 (BD Bioscience).

### 2.9. Statistical Analysis

All data were expressed quantitatively as the mean ± standard deviation. Paired Student's* t*-test for each pair of data and one-way analysis of variance was applied to compare the means. The *P* value of less than 0.05 was considered statistically significant. A statistical program for analysis (SPSS 13.0, SPSS Inc, Chicago, Illinois, USA) was used for all statistical analyses.

## 3. Results

### 3.1. Basic Clinical Characteristics of Patients

The basic clinical characteristics of patients were similar in very old patients (Table 1). 76 patients experienced heart failure of New York Heart Association Functional Class III-IV. The medical histories were similar and consistent with respect to the very old patients. The very old patients were discharged from the hospital on aspirin, clopidogrel, long-acting oral nitrates, ACEI/AT II blockers, beta blockers, and statins.

### 3.2. Plasma Levels of Circulating NO, eNOS, MDA, and SOD3 in Very Old Patients with Coronary Stenosis

The plasma levels of NO, eNOS, and SOD3 were decreased markedly in MVD with one CTO group when compared with MVD without CTO group and further reduced in MVD with multiple CTO group when compared with MVD with one CTO group (*P* < 0.001). Plasma levels of circulating MDA were significantly elevated in MVD with one CTO group compared to MVD without CTO group and further increased in MVD with multiple CTO when compared with MVD with one CTO group (*P* < 0.001) (Table 2).

### 3.3. Levels of NO, eNOS, MDA, SOD3, and New York Heart Association Functional Class of The Patients

The plasma levels of NO, eNOS, and SOD3 were decreased markedly in III group when compared with II group and further reduced in IV group when compared with III group (*P* < 0.001). Plasma levels of circulating MDA were significantly elevated in III group compared to II group and further increased in IV group when compared with III group (*P* < 0.001) (Table 3).

### 3.4. Levels of NO, eNOS, MDA, SOD3, and Left Ventricular Ejection Fraction of the Patients

The plasma levels of NO, eNOS, and SOD3 were decreased markedly in left ventricular ejection fraction group (30–39%) when compared with left ventricular ejection fraction group (40–55%) (*P* < 0.001) and further reduced in left ventricular ejection fraction group (<30%) when compared with left ventricular ejection fraction group (30–39%) (*P* < 0.001). Plasma levels of circulating MDA were significantly elevated in left ventricular ejection fraction group (30–39%) compared to left ventricular ejection fraction group (40–55%) and further increased in left ventricular ejection fraction group (<30%) when compared with left ventricular ejection fraction group (30–39%) (*P* < 0.001) (Table 4).

### 3.5. Effects of PECGGp on NO, eNOS, SOD3, MDA Syndecan-1, and Nrf2 Levels in Rat Cardiac Tissue of Infarcted Myocardium

In the rats with PECGGp-untreated model group, levels of NO, eNOS, and SOD3 in the cardiac muscle tissue were markedly decreased when compared with control group (*P* < 0.01), whereas levels of MDA in the cardiac muscle tissue were markedly elevated when compared with control group. Left ventricular ejection fraction was decreased significantly in Group B when compared with control group (*P* < 0.001).

The levels of NO, eNOS, and SOD3 were increased markedly in Group D when compared with Group B (*P* < 0.05, 0.01) and further increased in Group E when compared with Group D (*P* < 0.01). The levels of MDA were significantly declined in Group D compared to Group B and further decreased in Group E when compared with Group D (*P* < 0.01). Left ventricular ejection fraction was increased markedly in Group D when compared with Group B (*P* < 0.05) and further increased in Group E when compared with Group D (*P* < 0.01) (Table 5). The results indicated that PECGGp treatment had an effect of antioxidative damage and restored the antioxidative capacity in cardiac tissue of infarcted myocardium and the patient completely recovered from heart failure.

Cyclic GMP levels were significantly (*P* < 0.001) higher in PECGGp-treated group (2.7 ± 0.13 pmol/mg) compared to PECGGp-untreated group (0.53 ± 0.41 pmol/mg). The analysis showed higher expression of Nrf2 protein in PECGGp-treated group (35%, 17.40 ± 12.31) compared to PECGGp-untreated group (11%, 4.92 ± 4.57) (*P* < 0.001). Shedding of syndecan-1 was found to significantly increase in PECGGp-untreated group when compared with PECGGp-treated group (*P* < 0.001).

## 4. Discussion

Study showed that CTO was a frequent finding in very old patients, and primary percutaneous coronary intervention and coronary artery bypass graft surgery were used to treat CTO [43, 44]. The coronary artery disease patients who underwent primary percutaneous coronary intervention were mainly older adults. Results of the trials showed that primary percutaneous coronary intervention did not reduce the rate of major adverse cardiovascular events and may result in myocardial ischemic injury with elevating the rates of myocardial infarction recurrence and repeating coronary artery revascularizations in CTO patients. Coronary artery bypass graft surgery was more expensive and more invasive than primary percutaneous coronary intervention and performed in older patients with more severe coronary heart disease [27–30]. The results demonstrated that the long-term risk of cardiovascular death and adverse clinical outcome after coronary artery bypass grafting surgery were worse in older patients than in young patients [44]. Coronary angioplasty procedures took longer and had the risks of radiation-induced skin injury and acute renal failure. Coronary angioplasty procedures of coronary CTO involved important risk factors for coronary artery dissection and coronary perforation and pericardial tamponade [28]. Therefore, our study evaluated the strong potential as novel noninvasive therapy in very old patients with coronary CTO. It investigated the association of oxidant/antioxidant imbalance with coronary CTO in very old patients and demonstrated PECGGp as potential preventive and therapeutic agent.

The study showed that dysfunction of the NO signaling pathway increased risk of myocardial infarction. Increased reactive oxygen species levels inhibited NO bioactivity and production and led to coronary arterial injury. Cardiovascular disease often suppressed NO signaling pathway and augmented myocardial infarction risk [1–5]. Upregulation of NO levels can be a potential novel therapeutic target for inhibiting cardiac infarction and chronic congestive heart failure [5, 6]. The findings showed that reactive oxygen species inhibited the eNOS expression and activity in myocardium and the absence of eNOS led to myocardial infarction. Endothelial and myocyte overexpression of eNOS decreased atherosclerosis and influenced the pathophysiology of postmyocardial infarction. Cardiac myocyte-specific eNOS transgenic overexpression decreased oxidative stress and infarct area and improved cardiac function after coronary artery ligation. The clinical translation of potential regulator of eNOS expression may offer a potential new therapy for preventing myocyte imbalance between production and clearance of reactive oxygen species [9].

Our study has suggested that the levels of NO and eNOS were decreased markedly in MVD with one CTO group and further reduced in MVD with multiple CTO group. In the rats with PECGGp-untreated model group, levels of NO, eNOS, and SOD3 in cardiac tissue of infarcted myocardium were markedly decreased, whereas levels of MDA in the cardiac muscle tissue were markedly elevated. PECGGp increased the levels of NO and eNOS in cardiac tissue of infarcted myocardium and restored left ventricular ejection fraction, indicating key protective roles of NO and eNOS in myocardial infarction and heart failure. Our data showed that PECGGp had the potential for the prevention and therapy of coronary CTO in very old patients with heart failure.

Increased MDA levels contributed to enhancing the production of free radical and reduced the antioxidant activity. The suppression of reactive oxygen species generation or enhancement of endogenous antioxidant enzymes may decrease myocardial infarct size and improve myocardial dysfunction [11–14]. Several studies demonstrated that SOD3 was the most powerful antioxidant enzyme that defended the ischemic myocardium by inhibiting reactive oxygen species. The vascular SOD3 activity was severely reduced in coronary artery segments with stenoses of patients with coronary artery disease and SOD expression and activity being reduced in coronary atherosclerotic plaque. Further researches may lead to a novel antioxidant agent able either to increase SOD levels or to decrease MDA levels for myocardial infarction and chronic heart failure [19, 23–26]. We discovered that MVD with one CTO significantly elevated plasma levels of MDA and decreased SOD levels, and MVD with multiple CTO further increased MDA and reduced SOD levels. The MDA levels were significantly increased and levels of SOD were decreased markedly in cardiac tissue of infarcted myocardium and left ventricular dysfunction. PECGGp markedly increased levels of SOD and decreased MDA levels in cardiac tissue of infarcted myocardium and improved left ventricular ejection fraction.

It was concluded that expression of SOD3 in vivo did not directly affect atherosclerosis development. SOD3 was a key regulator of NO bioavailability in the blood vessel wall. Low vascular SOD3 expression played an important role in stenosis remodeling after injury, promoting oxidant stress and reduction in eNOS-derived NO bioavailability.

Increased MDA levels contributed to enhancing the production of free radical and reduced the antioxidant activity and elevating MDA levels were the main cause of myocardial infarction [8]. Atherosclerosis was associated with decreased NO bioactivity after MDA-mediated inhibition of the eNOS and SOD3 being critical in protecting NO from degradation. eNOS was a positive regulator for SOD3 expression and the reduction of eNOS contributed to decreased expression of SOD3 [9]. Our observations showed that the levels of NO, eNOS, and SOD3 were significantly decreased and MDA levels were markedly elevated in MVD patients with one CTO and further reduced and increased in MVD patients with multiple CTO. In the rats, levels of NO, eNOS, and SOD3 in cardiac tissue of infarcted myocardium were markedly decreased, whereas levels of MDA in the cardiac muscle tissue were markedly elevated. PECGGp restored the activities of antioxidant enzymes (eNOS and SOD3) and antioxidant (NO) and inhibited MDA in cardiomyocytes. NO generated within myocardium by the eNOS played a pivotal role in reduction of myocardial infarct size. SOD3 was the most powerful antioxidant enzyme that defended the myocardial infarction by inhibiting reactive oxygen species. The protective effects of PECGGp against oxidative injury were likely to be attributed to the upregulation of the endogenous cellular antioxidant system and MDA scavenging activity. The present study suggested that PECGGp had cardioprotective effects against myocardial infarction and heart failure, and the cardioprotective effects may be associated with increment of endogenous antioxidants, sustained antioxidant status in myocardial infarction, elevation of NO, eNOS, and SOD3 levels, and reduction in MDA levels.

Previous reports have shown that SOD3 derived cardiovascular injury recovery by increasing mitogenic signal transduction and reducing inflammation and apoptosis. The present reports have shown that SOD3 derives cardiovascular injury recovery by reducing inflammation and apoptosis.

Cardiovascular disease often suppressed NO signaling pathway and augmented myocardial infarction risk. Increased reactive oxygen species levels inhibited NO bioactivity and production and led to coronary arterial injury. The dysfunction of the NO signaling pathway increased risk of myocardial infarction. Upregulation of NO levels can be a potential novel therapeutic target for inhibiting cardiac infarction and chronic congestive heart failure [16, 17]. Endothelial and myocyte overexpression of eNOS decreased atherosclerosis and influenced the pathophysiology of postmyocardial infarction. The vascular SOD3 activity was severely reduced in coronary artery segments with stenoses of patients with coronary artery disease and SOD3 expression and activity were reduced in coronary atherosclerotic plaque. SOD3 was the most powerful antioxidant enzyme that defended the ischemic myocardium by inhibiting reactive oxygen species. Further researches may lead to a novel antioxidant agent able either to increase SOD3 levels or to decrease MDA levels for myocardial infarction and chronic heart failure [18–22]. PECGGp increased the levels of NO and eNOS in cardiac tissue of infarcted myocardium and restored left ventricular ejection fraction, indicating key protective roles of NO and eNOS in myocardial infarction and heart failure. PECGGp markedly increased levels of SOD3 and decreased MDA levels in cardiac tissue of infarcted myocardium and improved left ventricular ejection fraction. It was known that Ras-Erk pathway and G protein coupled receptor signal transduction increased SOD3 expression that then increased the healing of the injuries. Our results suggested that PECGGp may suppress unbalanced oxidant and antioxidant status in infarcted myocardium, coronary arteries damage, atherosclerotic plaque, and heart failure development by inhibiting levels of MDA and elevating NO, eNOS, and SOD3 levels. Ras-Erk mitogenic pathway and G protein coupled receptor signal transduction play significant roles in increasing SOD3 expression and further explain our observations of SOD3-mediated effects in injuries [45, 46].

The activation of the signaling pathway molecules upstream and downstream of SOD3 has been studied. The messenger molecule NO exerted its effects by the stimulation of NO sensitive guanylyl cyclase which led to enhanced production of the intracellular messenger cyclic GMP. Cyclic GMP protected endothelial progenitors from oxidative stress. Cyclic GMP levels were significantly elevated in PECGGp-treated group, thus providing evidence that PECGGp markedly increased levels of SOD3 and SOD3 expression elevated cyclic GMP levels in cardiac tissue of infarcted myocardium. The involvement of NO/cyclic GMP pathway in infarcted myocardium was confirmed [47, 48].

The nuclear factor erythroid 2-related factor (Nrf2) as the upstream regulator of SOD3 expression was a major antioxidant transcription factor to mediate the expression of antioxidant genes. Our results suggested that MDA can act via Nrf2 to regulate antioxidant defence gene expression. Nrf2 acted as a molecular sensor of cellular redox homeostasis disturbance and represented a powerful tool in SOD3 expression. SOD3 was regulated by the Nrf2 transcription factor and Nrf2 elevated SOD3 expression in cardiac tissue of infarcted myocardium. MDA affected the stability and the transcriptional functions of Nrf2. Notably, it was suggested that PECGGp activated SOD3 expression via Nrf2 pathway, protecting rat hearts against oxidative damage by decreasing MDA levels [49].

It has also been found that the loss of SOD3 may promote syndecan-1 vulnerable to oxidative stress and lead to syndecan-1 shedding and SOD3 inhibits oxidant-induced shedding of syndecan-1. Our results showed that the SOD3 decreased and shedding of syndecan-1 increased in cardiac tissue of infarcted myocardium. More importantly, The present study demonstrated that MDA contributed to syndecan-1 shedding in infarcted myocardium and that SOD3 protected the hearts against oxidative damage by limiting MDA-induced shedding of syndecan-1 [50].

Our results suggested that PECGGp may suppress unbalanced oxidant and antioxidant status in infarcted myocardium, coronary arteries damage, atherosclerotic plaque and heart failure development by inhibiting levels of MDA and elevating NO, eNOS, and SOD3 levels. These findings suggested that PECGGp may offer a potential new therapy for preventing and treating imbalance between production and clearance of reactive oxygen species in very old patients with MVD, with multiple CTO and heart failure.

## 5. Conclusion

The present study suggested that PECGGp had cardioprotective effects against myocardial infarction and heart failure, and the cardioprotective effects may be associated with increment of endogenous antioxidants, sustained antioxidant status in myocardial infarction, elevation of NO, eNOS, and SOD3 levels, and reduction in MDA. Therefore, PECGGp might be considered a potential therapeutic agent for preventing or treating CTO and heart failure in very old patients. However, more research is needed to study the clinical efficacy of PECGGp in prevention or treatment of CTO and heart failure, which may provide clinical evidence for a novel strategy in the prevention and therapy of unbalanced oxidant and antioxidant status and CTO in very old patients with heart failure.

## Figures and Tables

**Table 1:** Baseline characteristics of very old patients with coronary stenosis.

	CON	SVD	MVD without CTO	MVD with one CTO	MVD with multiple CTO
	*n* = 65	*n* = 114	*n* = 93	*n* = 71	*n* = 56
Age, years	85.9 ± 4.1	83.1 ± 3.1	86.2 ± 5.8	85.7 ± 5.1	87.7 ± 4.0
Male/ female	36/29	64/50	57/36	41/30	34/22
Hypertension	39/26	59/55	61/32	45/26	37/19
Hyperlipidemia	35/30	60/54	52/41	34/37	30/26
Smoking	55/10	72/42	82/11	64/7	50/6
Diabetes mellitus	45/20	70/44	53/40	43/28	36/20
Myocardial infarction	0/0	0/0	0/0	50/21	44/12
Smoker	48/17	90/24	82/11	61/10	50/6
Heart failure	0/0	0/0	7/3	23/10	25/8
Medications					
Aspirin	12/8	64/50	57/36	41/30	34/22
Beta blockers	0/0	67/47	59/34	45/26	37/19
ACEI/AT II blockers	0/0	65/49	57/36	41/30	34/22
Clopidogrel	0/0	63/51	60/33	50/21	36/20
Long-acting oral nitrates	0/0	69/45	59/34	41/30	34/22
Statins	0/0	64/50	57/36	40/31	36/20

CON: control; CTO: chronic total occlusion; MVD: multivessel disease; SVD: single vessel disease.

**Table 2:** Levels of Circulating NO, eNOS, MDA, and SOD3 in very old patients with coronary stenosis.

	CON	SVD	MVD without CTO	MVD with one CTO	MVD with multiple CTO
NO (*μ*mol/L)	61.2 ± 16.3	60.7 ± 17.1	51.4 ± 16.0	39.8 ± 15.9^*∗*^	19.6 ± 14.2^*∗∗*^
eNOS (pg/mL)	70.3 ± 25.1	71.5 ± 24.9	59.2 ± 22.4	42.6 ± 21.3^*∗*^	30.0 ± 19.6^*∗∗*^
MDA (nmol/L)	2.3 ± 1.4	2.0 ± 1.5	4.3 ± 1.7	6.1 ± 2.0^*∗*^	9.9 ± 2.9^*∗∗*^
SOD3 (U/mL)	19 ± 3.2	18.9 ± 4.1	15.2 ± 2.4	12.0 ± 2.1^*∗*^	6.1 ± 1.9^*∗∗*^

MDA: malondialdehyde; NO: nitric oxide; eNOS: endothelial nitric oxide synthase; SOD3: superoxide dismutase 3.

^*∗*^ *P* < 0.001 (MVD without CTO/MVD with one CTO). ^*∗∗*^ *P*< 0.001 (MVD with one CTO/MVD with multiple CTO).

**Table 3:** Levels of NO, eNOS, MDA, SOD3, and New York Heart Association Functional Class of The Patients.

	I	II	III	IV
NO (*μ*mol/L)	50.9 ± 16.3	52.0 ± 18.0	33.4 ± 15.7^*∗*^	18.1 ± 13.5^*∗∗*^
eNOS (pg/mL)	63.1 ± 27.8	60.9 ± 23.7	41.2 ± 19.5^*∗*^	22.7 ± 11.3^*∗∗*^
MDA (nmol/L)	2.9 ± 1.8	2.8 ± 1.9	5.1 ± 2.0^*∗*^	8.0 ± 2.7^*∗∗*^
SOD3 (U/mL)	20 ± 3.9	19.1 ± 4.0	11.9 ± 2.9^*∗*^	7.0 ± 2.3^*∗∗*^

MDA: malondialdehyde; NO: nitric oxide; eNOS: endothelial nitric oxide synthase; SOD3: superoxide dismutase 3.

^*∗*^ *P* < 0.001 (II/III). ^*∗∗*^ *P* < 0.001 (III/IV).

**Table 4:** Levels of NO, eNOS, MDA, SOD3, and LVEF of the patients.

LVEF (%)	≥55	40–55	30–39	<30
NO (*μ*mol/L)	59.8 ± 16.9	57.0 ± 14.1	30.7 ± 12.5^*∗*^	17.0 ± 10.0^*∗∗*^
eNOS (pg/mL)	69.1 ± 25.1	67.0 ± 23.8	47.2 ± 20.1^*∗*^	26.7 ± 17.6^*∗∗*^
MDA (nmol/L)	2.7 ± 1.9	2.9 ± 2.0	6.0 ± 2.3^*∗*^	8.9 ± 2.8^*∗∗*^
SOD3 (U/mL)	21.1 ± 3.9	19.9 ± 3.7	10.1 ± 3.1^*∗*^	6.2 ± 2.9^*∗∗*^

LVEF: left ventricular ejection fraction; MDA: malondialdehyde; NO: nitric oxide; eNOS: endothelial nitric oxide synthase; SOD3: superoxide dismutase 3.

^*∗*^ *P*< 0.001 (40–55%/30–39%). ^*∗∗*^ *P*< 0.001 (30–39%/<30%).

**Table 5:** Assessments of NO, eNOS, MDA, SOD3, and LVEF in postinfarct rat myocardium.

	Group A	Group B	Group C	Group D	Group E
LVEF (%)	55 ± 5.1	30 ± 3.0	56 ± 5.3	41 ± 4.9^*∗*^	57 ± 5.8^*∗∗∗*^
NO (nmol/mg prot)	20.5 ± 1.0	14.0 ± 0.3	21.3 ± 1.1	15.9 ± 0.5^*∗∗*^	18.7 ± 0.8^*∗∗∗*^
eNOS (U/mg prot)	15.3 ± 0.7	13.2 ± 0.1	16.1 ± 0.9	14.8 ± 0.6^*∗∗*^	16.0 ± 0.7^*∗∗∗*^
MDA(nmol/mg prot)	30.7 ± 23.8	60.3 ± 16.1	29.5 ± 12.9	49.9 ± 15.9^*∗*^	29.8 ± 14.1^*∗∗∗*^
SOD3 (U/mg prot)	50.9 ± 26.4	20.3 ± 14.7	51.7 ± 15.9	40.2 ± 15.0^*∗*^	53.1 ± 16.0^*∗∗∗*^

MDA: malondialdehyde; NO: nitric oxide; eNOS: endothelial nitric oxide synthase; SOD3: superoxide dismutase 3.

^*∗*^ *P* <0.01 (Group B/Group D). ^*∗∗*^ *P* < 0.05 (Group B/Group D). ^*∗∗∗*^ *P* < 0.01 (Group D/Group E).
